# Airborne pollen exposure and risk of hospital admission for allergic rhinitis in Beijing: A time‐stratified case‐crossover study

**DOI:** 10.1002/clt2.12380

**Published:** 2024-07-02

**Authors:** Yuhui Ouyang, Jun Yang, Jingxuan Zhang, Yun Yan, Shengzhi Sun, Jiajia Wang, Xiaobo Li, Rui Chen, Luo Zhang

**Affiliations:** ^1^ Department of Allergy Beijing Tongren Hospital Capital Medical University Beijing China; ^2^ Beijing Key Laboratory of Nasal Diseases Beijing Institute of Otolaryngology Beijing China; ^3^ Research Unit of Diagnosis and Treatment of Chronic Nasal Diseases Chinese Academy of Medical Sciences Beijing China; ^4^ School of Public Health Capital Medical University Beijing China; ^5^ Beijing Laboratory of Allergic Diseases Capital Medical University Beijing China

**Keywords:** airborne pollen, allergic rhinitis, time‐stratified case‐crossover

## Abstract

**Background:**

Airborne pollen is a crucial risk factor in allergic rhinitis (AR). The severity of AR symptoms can vary based on pollen type and concentration. This study aimed to estimate the association between exposure to different pollen types and AR risk.

**Methods:**

We obtained data from patients admitted to the Beijing Tongren Hospital for AR, and data on pollen concentration, meteorological factors, and fine particulate matter (PM_2.5_) from 13 districts in Beijing from 2016 to 2019. We used a time‐stratified case‐crossover study design and calculated odds ratios (ORs) related to the risk of AR associated with a 10 grain/1000 mm^2^ increase in total pollen concentrations for specific pollen types. A stratified analysis was conducted to assess whether the associations were varied by age and sex.

**Results:**

The OR of AR associated with a 10 grain/1000 mm^2^ increase in the 7‐day average pollen concentration was 1.014 (95% CI: 1.014, 1.015), 1.076 (95% CI: 1.070, 1.082), 1.024 (95% CI: 1.023, 1.025), 1.042 (95% CI: 1.039, 1.045), 1.142 (95% CI: 1.137, 1.147), 1.092 (95% CI: 1.088, 1.097), 1.046 (95% CI: 1.035, 1.058), and 1.026 (95% CI: 1.024, 1.028) for total pollen, Ulmus, Cupressaceae, Populus, Fraxinus, Pinus, Betula, and Artemisia, respectively. Both tree pollen (Ulmus, Cupressaceae, Populus, Fraxinus, Betula, and Pinus) and weed pollen (Artemisia, Chenopodium, and Humulus) were correlated with an increased risk of AR. These associations remained consistent across distinct subgroups defined by both age and sex.

**Conclusion:**

Exposure to pollen from trees and weeds might be associated with an increased risk of AR. This research provides valuable scientific support for both clinical practitioners and patients with AR regarding the hazards of pollen exposure.

## 1 BACKGROUND

Allergic rhinitis (AR) is an IgE‐mediated clinical condition characterized by watery rhinorrhea, nasal congestion, sneezing, and itching of the nose.^1^ The worldwide prevalence of AR has increased over the last 50 years, affecting between 10% and 30% of the world population,^2^, ^3^ particularly in developing countries.^4^ The International Study of Asthma and Allergies in Childhood, which surveyed children and adolescents from 98 countries covering a population of 130 million, reported AR prevalence of 8.5% and 14.6% in children aged 6–7 and 13–14 years, respectively.^5^ Moreover, AR notably impacts daily life by disrupting sleep patterns, resulting in reduced sleep duration, diminished sleep quality, and delayed sleep onset. These disturbances can lead to decreased productivity in work or school settings as well as depression and anxiety.^6^

Numerous factors contribute to an elevated risk of AR, including genetics, environmental triggers, and lifestyle.^7^ A study on the relationship between traffic‐related air pollution and AR has shown that AR prevalence is affected by the levels of SO_2_, NO_x_, O_3_ and particulate matter 10 μm or less in diameter (PM_10_).^8^ Airborne pollen is a crucial risk factor for AR; however, the underlying biological mechanisms are complex and multifaceted in nature.^9^ Airborne pollen contains allergenic proteins known as allergens, which on identification by the immune system, stimulate an allergic response.^10^ Notably, the severity of AR symptoms can vary based on factors such as pollen type and concentration, individual allergy susceptibility, and coexisting elements including exposure to other allergens or pollutants. For example, climate change causes an increase in the production of pollen and a change in the aspects increasing their allergenic properties. Through the effects of climate change, plant growth can be altered so that the new pollen produced are modified, affecting the severity of AR symptoms.^11^

Previous studies have examined the association between airborne pollen and the risk of AR.^12^, ^13^ For example, a time‐series study from 2002 to 2012 in New York, USA, found that increased levels of tree pollen in spring had significant associations with allergy medication sales and asthma‐related emergency department (ED) visits.^12^ Another nationwide study in the USA during 2008–2015 reported a wide discrepancy in airborne pollen types and health outcomes as well as a more pronounced increase in the AR risk for pollen from trees and weeds.^13^ Birch pollen is a major cause of AR in Europe. Over the last few decades, due to climate changes, levels of birch pollen have risen and the prevalence of birch pollen sensitization has also increased.^14^ Although the role of allergens in triggering AR symptoms is well known, evidence from epidemiological studies regarding the association between airborne pollen exposure and the risk of AR is still limited, especially for pollen from specific plant types, such as Ulmus, Cupressaceae, and Populus.

Accordingly, we conducted a time‐stratified case‐crossover study to examine the association between nine airborne pollen types (Ulmus, Cupressaceae, Populus, Fraxinus, Pinus, Betula, Artemisia, Chenopodium, and Humulus) on the basis of clinical significance and observed sensitization patterns in North China^15^ and hospital admissions for AR in Beijing, China, from 2016 to 2019. The findings of this research can be used to predict pollen hazards, which has implications for both clinical practitioners and patients with AR.

## 2 METHODS

### 2.1 Hospital admissions for AR

AR admissions were sourced from the Department of Otolaryngology‐Head and Neck Surgery at Tongren Hospital. As a comprehensive, state‐run, top‐tier Class Three Hospital, Tongren Hospital boasts a wide range of specialties supported by excellent clinical resources and expertise.

This retrospective study included patients admitted to Tongren Hospital for AR between March 1 and October 15 from 2016 to 2019. Patients who demonstrated symptom scores of ≥2 points for two or more nasal symptoms (sneezing, rhinorrhea, nasal itching and nasal obstruction) and sensitization to at least one of the allergens (tree pollen, grass pollen, weed pollen, molds, *blattella*, animal dander, house dust, and dust mites), as confirmed by the presence of specific immunoglobulin *E* (sIgE; ≥0.7 kUA/L) using ImmunoCAP system (Pharmacia, Uppsala, Sweden), were included. This study was approved by the Institutional Review Board of the Tongren Hospital and all participating patients with AR provided written informed consent.

### 2.2 Airborne pollen

**FIGURE 1:**
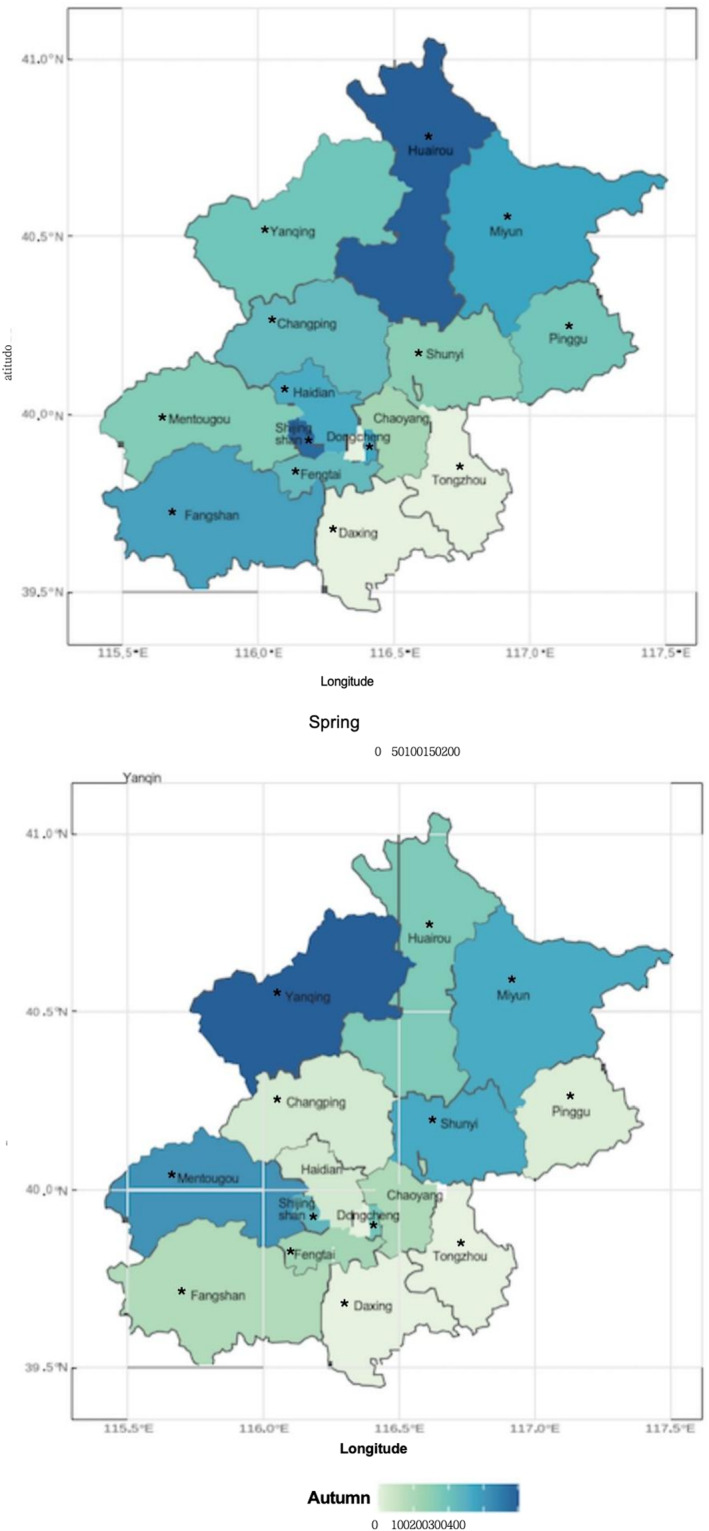
Geographic distribution of daily pollen concentrations (grains/1000 m^2^) at the 13 monitoring sites during different seasons in Beijing from 2016 to 2019.

Daily airborne pollen concentrations (grains/1000 mm^2^) were obtained from the Beijing Specialized Meteorological Observatory. Sampling was conducted at 13 sites distributed across 13 urban districts in Beijing; including Changping, Chaoyang, Fangshan, Fengtai, Dongcheng, Haidian, Huairou, Mentougou, Miyun, Pinggu, Shijingshan, Shunyi, and Yanqing by a gravitational monitoring (Figure 1). The 13 sampling sites were located close to residential areas and covered the majority of Beijing's districts (13 of 16), thereby representing general levels of pollen exposure in the city. These pollen samplers are placed on a roof 15–20 m above the ground, at least 1 m off the floor, and distant from any airflow barriers.

The final analysis included nine pollen genera^15^ with complete measurements: Ulmus (Elm), Cupressaceae (Cypress), Populus (Poplar), Fraxinus (Ash), Pinus (Pine), Betula (Birch) in Spring, and Artemisia (Mugwort), Chenopodium (Goosefoot), Humulus (Hop) in Autumn. The grass genus plants are not considered primary allergens in Beijing; therefore, pollen from grass is not specifically recorded in pollen data collection. The daily concentrations of each pollen type were calculated by averaging the daily concentrations across all sites. The pollen season for each taxon was determined based on the dates when accumulated pollen concentrations accounted for 2.5% and 97.5% of the annual pollen total.^16^ We defined the overall pollen season as the period spanning from the earliest start date to the latest end date for any pollen taxon within a year.

Daily ambient mean temperature (°C) and relative humidity data from March 1 to October 15, 2016–2019 were obtained from 13 monitoring stations in Beijing. We also obtained fine particulate matter (PM_2.5_) data from the China National Environmental Monitoring Center (https://data.cma.cn/).

### 2.3 Statistical analysis

A time‐stratified case‐crossover study design was used to determine the association between daily variations in distinct airborne pollen types and the risk of AR admission.^17^, ^18^, ^19^ In this design, patients acted as their own controls, with the analysis comparing daily pollen exposure on the event day to that on control days.^17^ Specifically, we defined the event day as the date of hospital admission for AR, and the control days as other days in the same year, month, and day of the week as the event day. Through self‐matching by the same month and year, the study design inherently controls for confounding factors that do not change markedly within a month. These variables include demographic characteristics, age, gender ratio, race, lifestyle factors, smoking status, pre‐existing diseases, allergic diseases, seasonality and long‐term patterns.^17^ We also controlled for time‐varying confounders in the analysis, which included daily average ambient temperature using a natural cubic spline with six degrees of freedom,^20^, ^21^ relative humidity using a natural cubic spline with three degrees of freedom,^20^, ^21^ PM_2.5_ levels, and public holidays. We used conditional logistic regression to estimate the odds ratios (ORs) associated with a 10 grain/1000 mm^2^ increase in each pollen type, considering one‐day, 4‐day, and 7‐day moving averages on event days. Assumptions of conditional logistic regression include a binary response variable, independence of observations, no extreme outliers, and a linear relationship between the predictor variables and the logit of the response variable. In the sensitivity analyses, we estimated the ORs for pollen types using 10‐ and 14‐day moving averages. We also performed subgroup analysis to examine the association variations among subgroups, defined by age (0–17, 18–64, ≥65 years) and sex (male vs. female).

Additionally, a general additive model was used to predict the relationship between pollen levels and hospital admissions for AR. For model fitting, a cubic regression spline was used to overcome the disadvantages of polynomial regression. The advantage of a cubic regression spline is its ability to fit non‐linear relationships. Compared to traditional linear regression, cubic regression splines can flexibly capture complex non‐linear relationships between variables. By fitting the data to multiple linear segments or cubic polynomial segments, cubic regression splines can more accurately approximate the true patterns in the data while avoiding excessive assumptions about the overall functional form. This flexibility makes cubic regression splines very useful for handling various types of data and analyzing complex relationships. Because seasonal differences were observed in this study, we predicted the dose–response relationships for total pollen levels and the dominant pollen types separately for spring and autumn. Spearman correlation was used to correlate spring and autumn pollen.

All statistical analyses were conducted with *R* software version 4.2.0 with the “gnm” package for case‐crossover data. *p* < 0.05 was considered to be statistically significant.

## 3 RESULTS

### 3.1 Descriptive characteristics

In total, 152,697 patients were diagnosed with AR during the study period. The mean number of patients admitted daily to the hospital attributing to AR was 195 between March 1 and October 15 from 2016 to 2019. No significant differences with respect to gender were observed (*p* > 0.05). For the age group, the average number of admissions was 37.5 (SD: 27.4) for 0–17 years, 139.7 (SD: 98.6) for 18–64 years, and 17.8 (SD: 11.8) for 65 years and over. The daily number of AR patients increased significantly in August‐September, consistent with a peak in Artemisia, Humulus, and Chenopodium pollen; however, this change was insignificant in spring with other pollen (Figure 2A–D). The median values of ambient temperature, relative humidity, and PM_2.5_ were 22.6°C, 55.9%, and 42 μg/m^3^, respectively (Table 1).

FIGURE 2Time‐series variations of the major pollen type concentrations (grains/1000 mm^2^) and daily number of allergic rhinitis patients during (A–D) spring and (E–H) autumn in Beijing for (A, E) 2016, (B, F) 2017, (C, G) 2018, and (D, H) 2019. Peak dates for tree pollen were March and April, whereas those for weed pollen were August and September.

**Figure:**
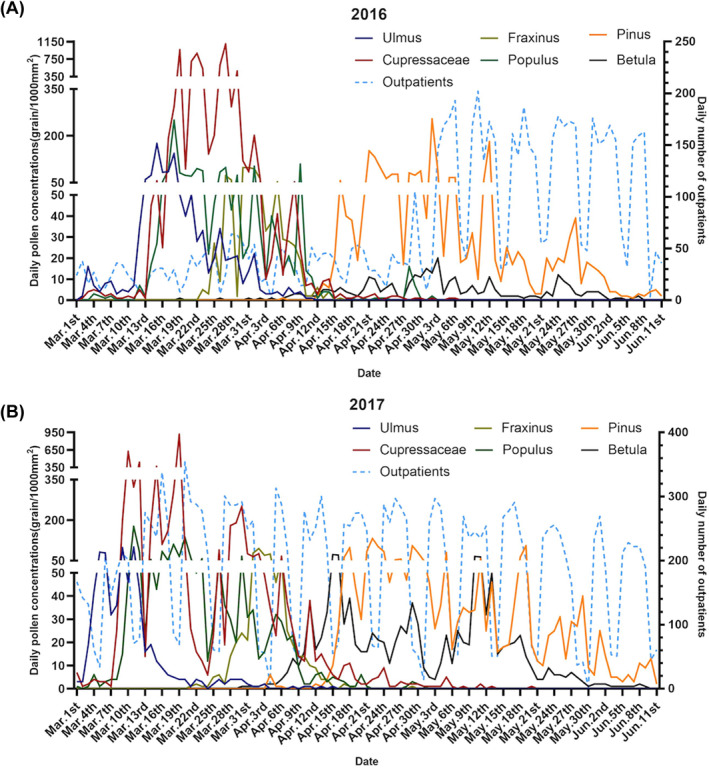

**Figure:**
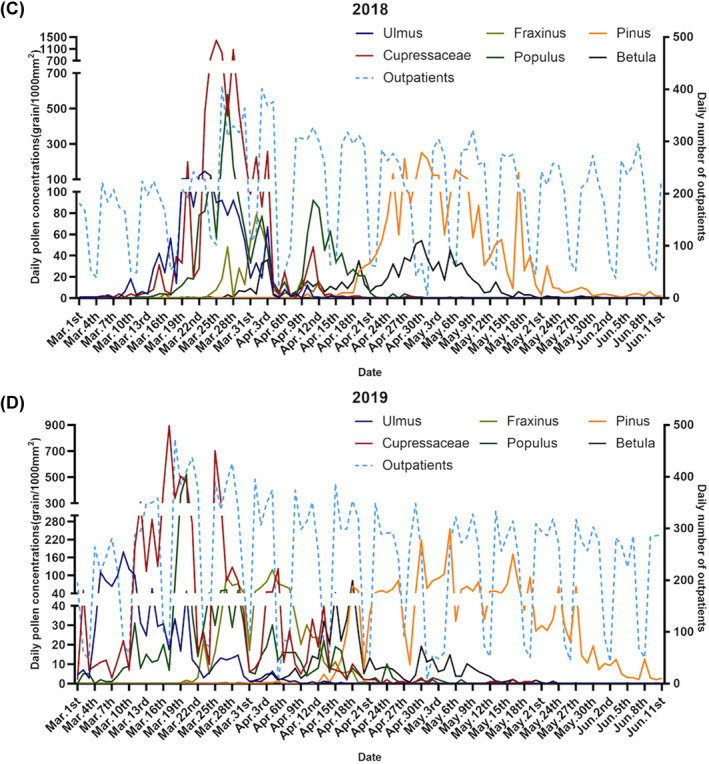

**Figure:**
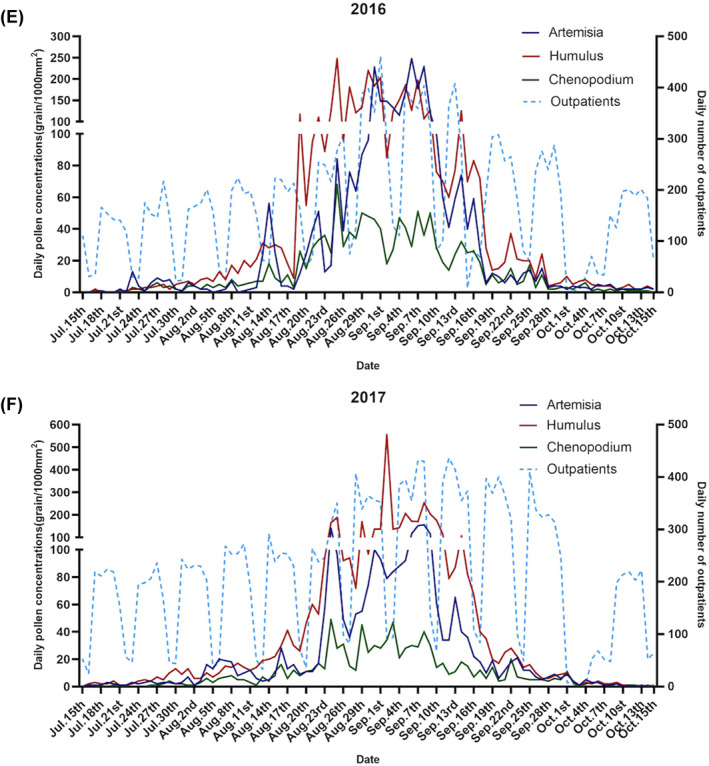

**Figure:**
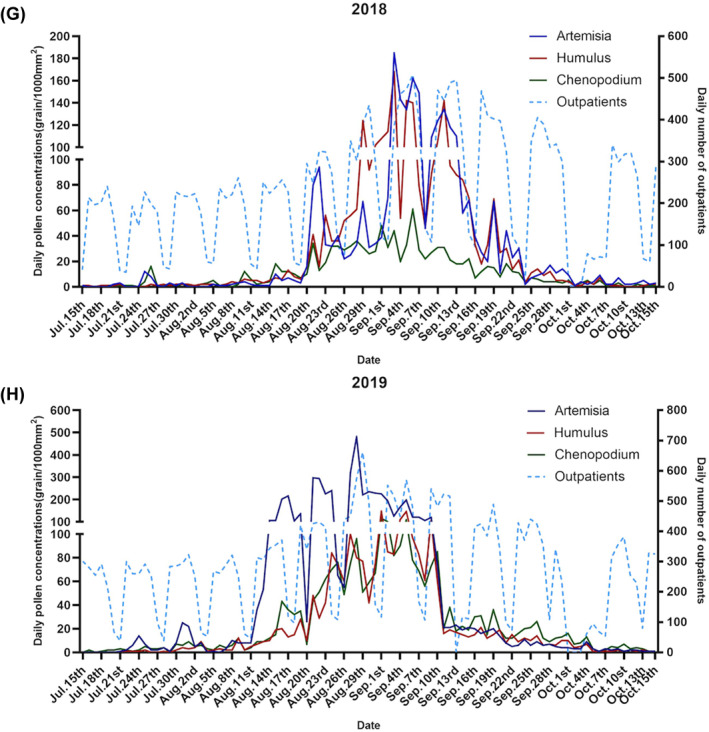

**TABLE 1:** Summary statistics of hospital admissions for allergic rhinitis, meteorological conditions, and air pollution during March 1–October 15, 2016–2019 in Beijing, China.

Variables	Mean	SD	Min	25th	50th	75th	Max
Allergic rhinitis							
Total (number/day)	195.0	125.9	1	66	202	282	662
Sex							
Male (number/day)	98.9	63.0	0	34	102	143	329
Female (number/day)	96.1	63.9	0	32	99	138	333
Age (years)							
0–17 (number/day)	37.5	27.4	0	15	32	54	170
18–64 (number/day)	139.7*	96.6	0	45	133	206	458
65+ (number/day)	17.8	11.8	0	6	19	27	56
Meteorological conditions							
Ambient temperature (°C)	21.6	6.5	1	18	23	26	32
Relative humidity (%)	55.2	19.4	10	40	56	72	96
Air pollutant							
PM_2.5_ (μg/m^3^)	50.8	38.9	4	23	42	68	340

Abbreviations: min, minimum; max, maximum; 25^th^, 50^th^ and 75^th^: the 25th, 50th, 75th percentiles; PM_2.5_, fine particulate matter; SD, standard deviation.

*p < 0.05.

### 3.2 Characteristics of pollen types

The four types of early spring tree pollen lasted from March 1 to April 18 and included Cupressaceae, Populus, Ulmus, and Fraxinus, accounting for 25.62%, 7.16%, 4.44%, and 2.77% of the average annual pollen count, respectively (Table S1). The duration of the two late‐spring tree pollen grains, Pinus and Betula, ranged from April 16 to May 26, accounting for 9.79% and 2.83% of the average annual pollen count, respectively (Figure 2A–D, Table S1). Weed pollen (Artemisia, Chenopodium, and Humulus) durations ranged from August 1 to October 7, accounting for 13.14%, 13.33% and 5.40% of the average annual pollen count, respectively (Table S1). The 4‐year average peak dates for tree pollen were March 10 to April 5, whereas those for weed pollen were August 14 to September 15 (Figure 2E–H).

### 3.3 Correlation statistics

**FIGURE 3:**
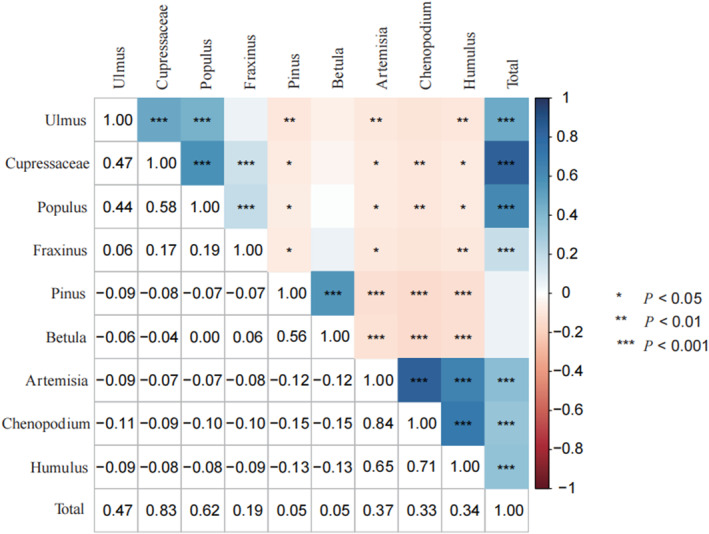
Correlation matrix of average pollen concentrations of different pollen types in Beijing. Airborne pollen types within cluster two were positively correlated. Cluster one includes Ulmus, Cupressaceae, Populus, Fraxinus, Pinus, and Betula; cluster two includes Artemisia, Chenopodium, and Humulus. ***p < 0.001, **p < 0.01, *p < 0.05.

In the Figure 3, we found a stronger association between Chenopodium and Artemisia (*r* = 0.84), Humulus and Chenopodium (*r* = 0.71) and found a moderate association of Cupressaceae with Ulmus (*r* = 0.47) and Populus (*r* = 0.58), Betula and Pinus (*r* = 0.56), and Humulus and Artemisia (*r* = 0.65).

### 3.4 Association between different pollen types and risk of allergic rhinitis

The general additive model showed the estimated cumulative rate ratio for AR risk with a 10 grain/1000 mm^2^ increase in each pollen type. During the 0–1 lag days, 0–4 lag days, and 0–7 lag days of average pollen, a 10 grain/1000 mm^2^ increase in all types of airborne pollen concentrations was associated with a higher risk of AR (Table 2). For example, a 10 grain/1000 mm^2^ increase in Fraxinus was associated with an OR of 1.185 (95% CI:1.178, 1.193) for AR, and the pronounced effects were also detected during lag 0–4 days (OR: 1.253, 95% CI: 1.244, 1.262) and during lag 0–7 days (OR: 1.142, 95% CI: 1.137, 1.147). Similar results were also observed during the 0–10 and 0–14 lag days (Table S2).

**TABLE 2:** Odds ratio of allergic rhinitis associated with a 10 grain/1000 mm^2^ increase in pollen types over multiple lag day durations during the pollen season in Beijing, 2016–2019.

Pollen type	0–1 lag days	0–4 lag days	0–7 lag days
	OR (95% CI)	OR (95% CI)	OR (95% CI)
Total	1.013 (1.013, 1.013)	1.014 (1.014, 1.015)	1.014 (1.014, 1.015)
Ulmus	1.021 (1.017, 1.026)	1.027 (1.022, 1.032)	1.076 (1.070, 1.082)
Cupressaceae	1.020 (1.019, 1.021)	1.024 (1.023, 1.025)	1.024 (1.023, 1.025)
Populus	1.027 (1.025, 1.029)	1.043 (1.040, 1.046)	1.042 (1.039, 1.045)
Fraxinus	1.185 (1.178, 1.193)	1.253 (1.244, 1.262)	1.142 (1.137, 1.147)
Pinus	1.045 (1.042, 1.048)	1.077 (1.073, 1.081)	1.092 (1.088, 1.097)
Betula	1.006 (0.997, 1.016)	1.018 (1.008, 1.029)	1.046 (1.035, 1.058)
Artemisia	1.029 (1.027, 1.031)	1.029 (1.027, 1.031)	1.026 (1.024, 1.028)
Chenopodium	1.087 (1.080, 1.093)	1.059 (1.051, 1.066)	1.048 (1.038, 1.059)
Humulus	1.043 (1.040, 1.045)	1.016 (1.013, 1.019)	1.005 (1.001, 1.008)

*Note*: Models were adjusted for ambient temperature, relative humidity, public holiday, and PM_2.5_.

### 3.5 Stratified analysis

To identify susceptible subpopulations, we conducted stratified analysis to examine whether the association varied among subgroups defined by sex and age. The risk of AR visits showed strong age‐dependency (Table 3). The 18–64 age group showed the strongest associations (e.g., OR of 1.055 [95% CI: 1.042, 1.068] in the 18–64 age group compared to 1.024 [95% CI: 0.989, 1.060] in individuals aged ≥65 years and 1.018 [95% CI: 0.989, 1.049] in the 0–17 age group associated with a 10 grain/1000 mm^2^ increase in Betula pollen); however, no associations with sex were revealed (1.014 (95% CI: 1.014, 1.015) for males and 1.014 (95% CI: 1.014, 1.015) for females).

**TABLE 3:** Odds ratio of allergic rhinitis associated with a 10 grain/1000 mm^2^ increase in pollen types by sex and age over 0–7 lag days during the pollen season in Beijing, 2016–2019.

Pollen type	OR and 95% CI (stratified by gender)		OR and 95% CI (stratified by age (year))		
	Male	Female	0–17	18–64	≥65
Total	1.014 (1.014, 1.015)	1.014 (1.014, 1.015)	1.014 (1.013, 1.016)	1.015 (1.014, 1.016)	1.010 (1.008, 1.011)
Ulmus	1.076 (1.068, 1.085)	1.076 (1.067, 1.084)	1.077 (1.059, 1.095)	1.077 (1.071, 1.084)	1.072 (1.052, 1.092)
Cupressaceae	1.024 (1.022, 1.025)	1.024 (1.023, 1.026)	1.023 (1.020, 1.025)	1.025 (1.024, 1.026)	1.019 (1.016, 1.022)
Populus	1.039 (1.035, 1.043)	1.045 (1.042, 1.049)	1.034(1.025, 1.043)	1.045 (1.041, 1.048)	1.031 (1.021, 1.040)
Fraxinus	1.138 (1.131, 1.145)	1.146 (1.139, 1.153)	1.119 (1.104, 1.133)	1.145 (1.139, 1.151)	1.145 (1.129, 1.162)
Pinus	1.094 (1.088, 1.101)	1.090 (1.084, 1.097)	1.081 (1.070, 1.093)	1.096 (1.091, 1.101)	1.083 (1.069, 1.098)
Betula	1.054 (1.038, 1.070)	1.039 (1.023, 1.055)	1.018 (0.989, 1.049)	1.055 (1.042, 1.068)	1.024 (0.989, 1.060)
Artemisia	1.026 (1.023, 1.028)	1.026 (1.024, 1.029)	1.022 (1.018, 1.026)	1.029 (1.027, 1.031)	1.011 (1.005, 1.018)
Chenopodium	1.060 (1.051, 1.070)	1.061 (1.052, 1.071)	1.056 (1.041, 1.071)	1.066 (1.058, 1.074)	1.019 (0.996, 1.043)
Humulus	1.019 (1.015, 1.023)	1.017 (1.013, 1.021)	1.015 (1.008, 1.022)	1.020 (1.017, 1.024)	1.002 (0.993, 1.011)

*Note*: Models were adjusted for ambient temperature, relative humidity, public holidays, and PM_2_.

**FIGURE 4:**
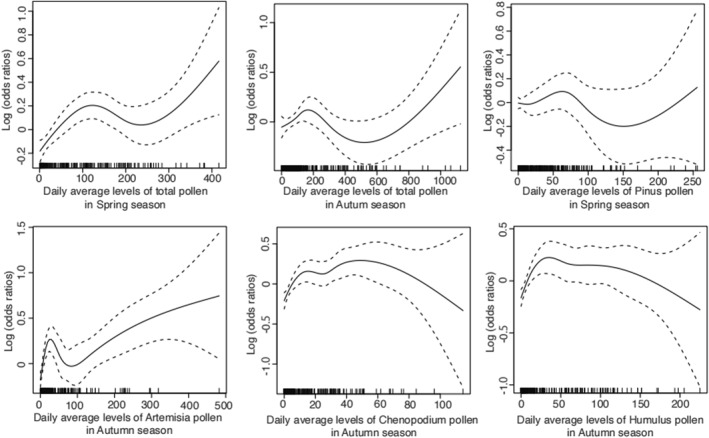
Association between exposure to different pollen types and risk of AR. (A) Association between exposure to spring pollen and risk of AR. Daily average levels of total spring pollen reached approximately 200 grains/1000 mm^2^. The number of AR hospital admissions increased to a moderate peak and then decreased until pollen levels reached 600 grains/1000 mm^2^, after which the number of hospital visits increased again with an increase in pollen levels. (B) Association between exposure to autumn pollen and risk of AR. In autumn, the first peak of hospital admissions appeared at approximately 100 grains/1000 mm^2^. When total pollen levels exceeded 200 grains/1000 mm^2^, the number of AR hospital admissions increased steadily with increasing levels of total autumn pollen. (C) Pinus pollen showed dose–response curves similar to total pollen in spring. (D) Artemisia pollen showed dose–response curves similar to total pollen in autumn. (E, F) Chenopodium and Humulus pollen contributions to AR hospital admissions decreased once pollen levels exceeded 50 grains/1000 mm^2^. Spring pollen: from March 1 to May 26 and Autumn pollen: from July 28 to October 7. AR, allergic rhinitis.

We observed similar dose–response patterns with different thresholds in spring and autumn (Figure 4A–F). In spring, when the daily average levels of total pollen reached approximately 200 grains/1000 mm^2^, the number of AR hospital admissions showed a minor peak before decreasing until the levels of total pollen reached 600 grains/1000 mm^2^, after which the number of hospital visits increased again following a surge in pollen levels (Figure 4A). However, in autumn, the first peak in hospital admissions appeared at approximately 100 grains/1000 mm^2^, and when the total pollen levels exceeded 200 grains/1000 mm^2^, the number of AR hospital admissions increased steadily (Figure 4B). The dose–response patterns also differed according to the pollen type. In spring, the curves were similar for Pinus and total pollen (Figure 4C). However, of the dominant pollen types in autumn, Artemisia contributed the most to AR hospital admissions (Figure 4D), whereas the contributions of Chenopodium and Humulus decreased once the pollen levels exceeded 50 grains/1000 mm^2^ (Figure 4E,F).

## 4 DISCUSSION

In this time‐stratified case‐crossover study of 152,697 AR cases from 2016 to 2019 in Beijing, China, we assessed the relationship between exposure to various types of airborne pollen and the risk of hospital admission for AR. Our findings indicated that tree pollen (Ulmus, Cupressaceae, Populus, Fraxinus, Betula, and Pinus) and weed pollen (Artemisia, Chenopodium and Humulus) are associated with an increased risk of AR. The 18–64 age group showed the strongest associations between pollen exposure and hospital visits, especially Fraxinus pollen, whereas no sex‐dependent association was found. We also observed different threshold levels for total pollen during spring and autumn.

Evidence of an association between types of airborne pollen and AR admissions is limited and inconclusive.^12^, ^13^, ^22^, ^23^ In this study, we found that the daily number of AR patients increased significantly in August‐September, consistent with a peak of pollen concentration; however, this change was insignificant in spring, which was related to the wide variety of pollen, inconsistent flowering times, and two peak pollen periods in spring. In addition, the relocation of the hospital in the spring of 2016 had a certain impact on the number of outpatients. Although the role of allergens in triggering AR symptoms is well known, the effect of pollen varies according to environmental factors, such as meteorological factors and environmental pollution.^11^ Specifically, the concentrations and sensitization of pollen dispersed in the air vary depending on the average temperature and relative humidity. Three dominant types of pollen are associated with AR: tree, grass, and weed.^12^, ^13^ A recent large‐scale national study in the USA suggested that spring pollen from maple, birch, beech, ash, oak, and sycamore showed significant associations with Asthma‐related ED visits and allergy medication sales.^12^ Another study used a random effects meta‐analysis to produce an overall risk estimate for each pollen type and AR‐related visits and found that the relative risk of AR‐related visits increased as tree, grass and weed pollen concentrations increased.^13^ A cross‐sectional study in the grasslands of northern China suggested that Artemisia, Chenopodium, and Humulus scandens were the most common and dominant pollen allergens.^24^ Another retrospective study from Beijing reported that the risk of hospital admissions for AR among children was elevated upon exposure to tree pollen such as pine, cypress, and poplar^23^, which agrees with the findings of this study. In addition, the increase in outpatient visits around the peak of the weed pollen season may be related to fungal spores. Fungal spores, a potent source of known aeroallergens, are disseminated in warm air, with a peak during the late summer and early autumn. A study suggests that the combination of mold spores and increased pollen allergen exposure during thunderstorms may be responsible for severe asthma.^25^ Unfortunately, airborne mold concentrations were not monitored in this study.

We also found that adults aged 18–64 years are more likely to be affected by pollen exposure. Previous studies have found similar trends among different‐aged patients with AR; for example, a study conducted in Korea investigated the age distribution of 2883 patients with AR between 2003 and 2014 and showed that AR prevalence in the population progressively decreased with age.^26^ Our results indicated that age differentiation occurs between patients younger than 18 years and elderly people, highlighting adults aged 18–64 as a potential group for pollen exposure, especially those exposed to pollen from Fraxinus.

The biological mechanisms behind the association between pollen exposure and AR are diverse and complex, including human immune disorders, composition and properties of pollen allergens, length of pollen allergen exposure, and environmental factors.^27^, ^28^, ^29^ Pollens are a source of a group of molecules containing proteins (including enzymes), lipids, and polysaccharides.^30^ These components interact with each other, resulting in inflammation. The main allergens from tree and weed pollen are proteins belonging to Bet v1 homologs, lipid transfer proteins, profilins, polcalcins, and *β*‐expansins.^31^, ^32^ Exposure to pollen can trigger allergic sensitization through direct stimulation of antigen‐presenting cells and in sensitized individuals via cross‐linkage of allergen bound specific IgE via the high‐affinity Fc_ε_ receptor.^33^, ^34^, ^35^

This study has several limitations. First, we included outpatients only for patients with AR admitted to a single hospital in Beijing, China. We plan on conducting a multi‐center study, which will extend this study to other cities. Second, we used airborne pollen concentrations as a proxy for personal pollen exposure, which may have introduced misclassification bias. However, such a misclassification bias would likely have biased our findings toward a null association.^36^ Third, pollen concentrations were identified and counted manually, which induced a certain level of manual error. Nevertheless, this study revealed that different thresholds of total pollen corresponded to the first peak of AR‐related hospital admissions in both seasons. These results can provide valuable scientific support for clinical practitioners and patients with AR and help reduce pollen hazards.

## 5 CONCLUSION

In this time‐stratified case‐crossover study comprising 152,697 patients with AR admitted to the Beijing Tongren Hospital, China, we found that tree pollen (Ulmus, Cupressaceae, Populus, Fraxinus, Betula, and Pinus) and weed pollen (Artemisia, Chenopodium, and Humulus) are associated with an increased risk of AR admission. Our findings provide important insights into addressing pollen hazards and reducing the risk of AR.

## AUTHOR CONTRIBUTIONS

**Yuhui Ouyang**: Conceptualization; data curation; formal analysis; investigation; methodology; writing – original draft; writing – review & editing. **Jun Yang**: Data curation; investigation. **Jingxuan Zhang**: Data curation; formal analysis. **Yun Yan**: Data curation; formal analysis. **Shengzhi Sun**: Data curation. **Jiajia Wang**: Formal analysis. **Xiaobo Li**: Formal analysis. **Rui Chen**: Conceptualization; writing – review & editing. **Luo Zhang**: Conceptualization; funding acquisition; investigation; methodology; project administration; writing – original draft; writing – review & editing.

## CONFLICT OF INTEREST STATEMENT

The authors declare that they have no conflicts of interest.

## Supporting information

Supporting Information S1

## Data Availability

Data available on request from the authors.
