# Supplementary material for: Airborne pollen exposure and risk of hospital admission for allergic rhinitis in Beijing: A time‐stratified case‐crossover study
Source: Clin Transl Allergy. 2024 Jul 2;14(7):e12380. doi: 10.1002/clt2.12380 (PMC11220181; doi:10.1002/clt2.12380)
Supplement: Supplementary file 1 — Supporting Information S1 [file CLT2-14-e12380-s001.docx]

Supplementary Table 1: Main pollen species and proportion in Beijing from 2016 to 2019

|  | **Ulmus** | **Cupressaceae** | **Populus** | **Fraxinus** | **Pinus** | **Betula** | **Artemisia** | **Humulus** | **Chenopodium** |
| --- | --- | --- | --- | --- | --- | --- | --- | --- | --- |
|  | **pollen concentration (%)** | **pollen concentration (%)** | **pollen concentration (%)** | **pollen concentration (%)** | **pollen concentration (%)** | **pollen concentration (%)** | **pollen concentration (%)** | **pollen concentration (%)** | **pollen concentration (%)** |
| 2016 | 1127  (4.22) | 7368  (27.58) | 1721  (6.44) | 771  (2.89) | 2326  (8.71) | 310  (1.16) | 3135  (11.74) | 4332  (16.22) | 1278  (4.78) |
| 2017 | 697  (2.73) | 5236  (20.53) | 1602  (6.28) | 627  (2.46) | 2223  (8.72) | 1167  (4.58) | 2426  (9.51) | 4659  (18.27) | 932  (3.66) |
| 2018 | 1554  (6.33) | 7099  (28.93) | 2352  (9.59) | 446  (1.82) | 2867  (11.69) | 901  (3.67) | 2640  (10.76) | 2649  (10.80) | 1089  (4.44) |
| 2019 | 1206  (4.46) | 5908 (25.45) | 1727  (6.39) | 1061  (3.92) | 2712  (10.03) | 514  (1.90) | 5554  (20.54) | 2173  (8.04) | 2361  (8.73) |
| Average | 1146  (4.44) | 6403  (25.62) | 1851  (7.17) | 726  (2.77) | 2532  (9.79) | 723  (2.83) | 3439  (13.14) | 3453  (13.33) | 1415  (5.40) |

Supplementary Table 2. Odds ratio of allergic rhinitis associated with a 10 grain/1,000 mm^2^ increase in pollen types across 0–10 and 0–14 lag days during the pollen season in Beijing, 2016–2019.

| **Pollen type** | **0–10 lag days** | **0–14 lag days** |
| --- | --- | --- |
|  | OR (95% CI) | OR (95% CI) |
| Total | 1.014 (1.013, 1.014) | 1.014 (1.013, 1.014) |
| Ulmus | 1.105 (1.099, 1.112) | 1.172 (1.162, 1.182) |
| Cupressaceae | 1.025 (1.024, 1.026) | 1.038 (1.037, 1.040) |
| Salicaceae | 1.033 (1.030, 1.036) | 1.049 (1.044, 1.053) |
| Fraxinus | 1.401 (1.385, 1.416) | 1.561 (1.539, 1.581) |
| Pinus | 1.102 (1.097, 1.106) | 1.131 (1.125, 1.137) |
| Betula | 1.087 (1.075, 1.099) | 1.089 (1.075, 1.102) |
| Artemisia | 1.024 (1.022, 1.025) | 1.024 (1.022, 1.026) |
| Chenopodium  Humulus | 1.029 (1.017, 1.041)  0.999 (0.995, 1.003) | 1.000 (0.999, 1.000)  0.995 (0,993, 1.000) |

Models were adjusted for ambient temperature, relative humidity, public holiday, and PM_2.5_
